# Biochemical characterisation of a PL24 ulvan lyase from seaweed-associated *Vibrio* sp. FNV38

**DOI:** 10.1007/s10811-023-03136-3

**Published:** 2023-12-07

**Authors:** Valerie J. Rodrigues, Diane Jouanneau, Narcis Fernandez-Fuentes, Lucy A. Onime, Sharon A. Huws, Annamma A. Odaneth, Jessica M. M. Adams

**Affiliations:** 1 Institute of Biological, Environmental and Rural Sciences, Aberystwyth University, Gogerddan, Aberystwyth, SY23 3EE United Kingdom; 2 DBT-ICT Centre for Energy Biosciences, Institute of Chemical Technology, Nathalal Parekh Marg, Matunga (East), Mumbai, 400019 Maharashtra India; 3 Laboratory of Integrative Biology of Marine Models (LBI2M), Station Biologique de Roscoff (SBR), CNRS, 29688 Roscoff, Bretagne France; 4 Laboratory of Integrative Biology of Marine Models (LBI2M), Station Biologique de Roscoff (SBR), Sorbonne Université, Roscoff, Bretagne, France; 5 Institute for Global Food Security, Queen’s University Belfast, 19 Chlorine Gardens, Belfast, BT9 5DL United Kingdom

**Keywords:** Macroalgae, Polysaccharides, Enzymes, Ulvan, *Vibrio*

## Abstract

Ulvan is a green macroalgal cell wall polysaccharide that has tremendous potential for valorisation due to its unique composition of sulphated rhamnose, glucuronic acid, iduronic acid and xylose. Several potential applications such as production of biofuels, bioplastics and other value-added products necessitate the breakdown of the polysaccharide to oligomers or monomers. Research on ulvan saccharifying enzymes has been continually increasing over the last decade, with the increasing focus on valorisation of seaweed biomass for a biobased economy. Lyases are the first of several enzymes that are involved in saccharifying the polysaccharide and several ulvan lyases have been structurally and biochemically characterised to enable their effective use in the valorisation processes. This study investigates the whole genome of *Vibrio* sp*.* FNV38, an ulvan metabolising organism and biochemical characteristics of a PL24 ulvan lyase that it possesses. The genome of *Vibrio* sp*.* FNV38 has a diverse CAZy profile with several genes involved in the metabolism of ulvan, cellulose, agar, and alginate. The enzyme exhibits optimal activity at pH 8.5 in 100 mM Tris–HCl buffer and 30 °C. However, its thermal stability is poor with significant loss of activity after 2 h of incubation at temperatures above 25 °C. Breakdown product analysis reveals that the enzyme depolymerised the polysaccharide predominantly to disaccharides and tetrasaccharides.

**Supplementary Information:**

The online version contains supplementary material available at 10.1007/s10811-023-03136-3.

## Introduction

**Fig. 1:**
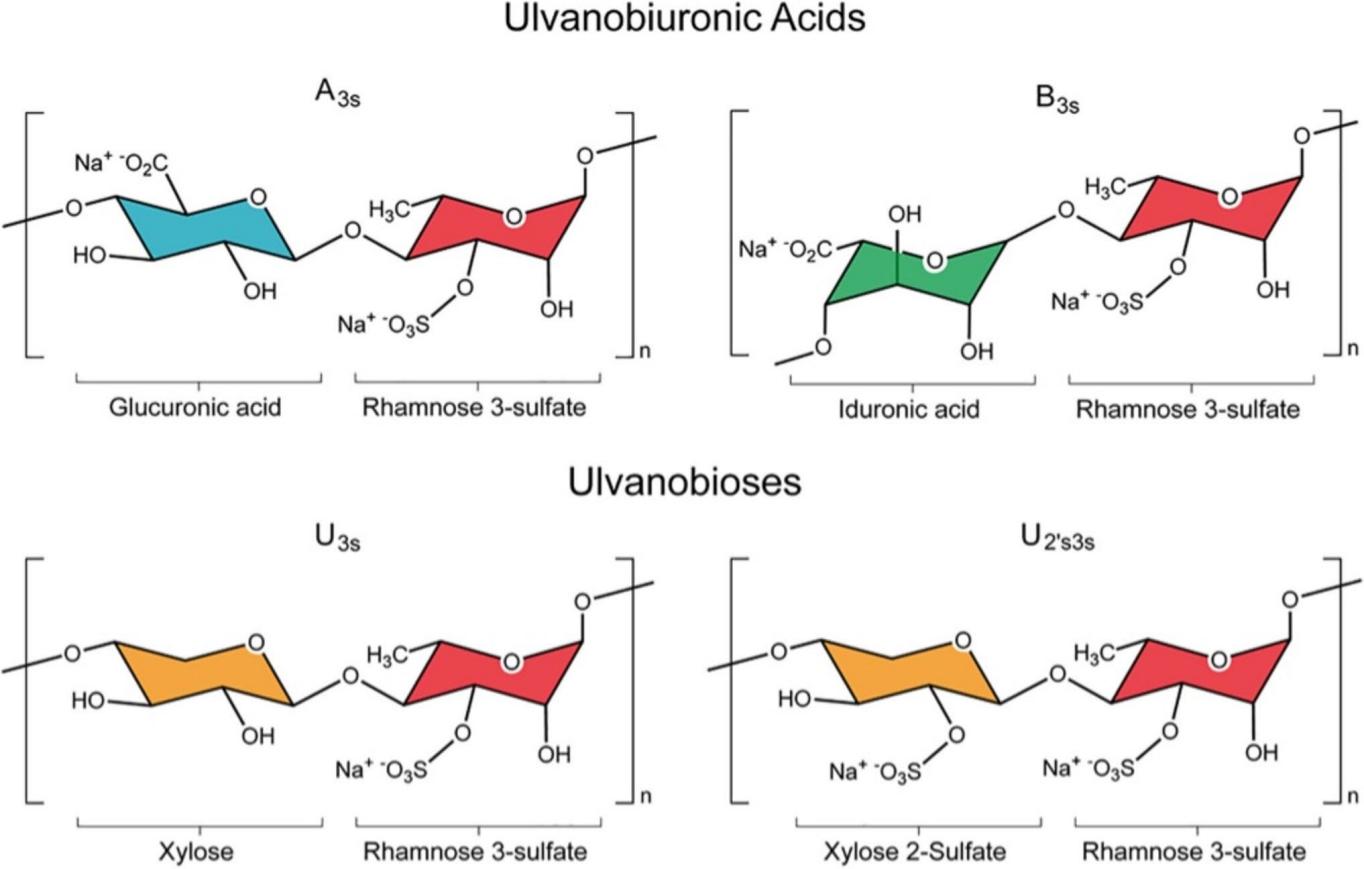
Main disaccharide units within ulvan. Reproduced from (Kidgell et al. 2019). Glucuronic acid (blue), rhamnose 3-sulphate (red), iduronic acid (green), xylose (orange). Xylose can also contain a sulphate group, as seen in U2’S,3S

Macroalgae are receiving increasing attention as a sustainable feedstock for bio-based products such as biochemicals, bioplastics and biofuels as the world seeks to move towards a biobased economy. Factors that contribute to the attractiveness of macroalgae as a feedstock include their carbon assimilation potential and their ability to quickly proliferate without any requirements for land, fresh water or nutrients (Kaladharan et al. 2009; Adams et al. 2017). Additionally, increasing occurrences of uncontrolled macroalgal blooms called gold and green tides result in the generation of enormous amounts of biomass that could be exploited for industrial applications. Green seaweed belonging to the genus *Ulva* are of particular interest because they are the primary seaweed responsible for green tides around the world and the main or only species in 52% of recorded blooms up until 2019 (Smetacek and Zingone 2013; Joniver et al. 2021). They are a rich source of carbohydrates with around 8–30% dry weight consisting of ulvan, an anionic, sulphated water-soluble polysaccharide predominantly composed of rhamnose, glucuronic acid, iduronic acid and xylose (Lahaye and Robic 2007). The backbone is made of these monosaccharides through α and β-(1,4)-linkages in repeating disaccharide units as shown in Fig. 1 (Kidgell et al. 2019), with 1,2 and 1,3 linkages also present (Lahaye 1998). The main disaccharides are ulvanobiuronic acids (aldobiuronic acids) types A and B; with ulvanobioses (disaccharide aldobioses) in type U also present but at much lower levels. Ulvanobiuronic acid type A_3S_, consist of a β-D-glucuronic acid (1,4)-linked to an α-L-rhamnose 3-sulphate; whereas type B_3S_ has an α-L-iduronic acid (1,4)-linked to an α-L-rhamnose 3-sulphate. Ulvanobiose U_3S_ and U_2’S,3S_ consist of a β-D-xylose or a β-D-xylose 2-sulphate respectively (1,4)-linked to α-L-rhamnose 3-sulphate moieties (Kidgell et al. 2019). There are differences in proportions and overall ulvan composition between *Ulva* species and through cultivation conditions, making the exact composition diverse (Tang et al. 2021).

Saccharification of ulvan relies on a variety of enzymes to hydrolyse all bonds but enables utilisation of the monosugars to produce industrially important products and chemicals. These include fermentation products 1,2-propanediol and lactic acid as well as biofuels such as ethanol and butanol (Saxena et al. 2010; Hwang et al. 2012; Diallo et al. 2018). Rhamnose can be used as a precursor for the synthesis of furaneol, creating a strawberry aroma and used extensively in the food, beverage, healthcare and pharmaceutical sectors; it is also used as an anti-aging agent in cosmetics (Kuivanen and Richard 2016). Iduronic acid can also be used for the synthesising of fragment analogues of heparin. Heparin is a common anticoagulant drug which greatly enhances the effect of the inhibitor antithrombin, by improving binding with target enzymes, making this a valuable medical requirement (Miyazawa et al. 2023). Iduronic acid can also be produced though chemical synthesis but this is a lengthy and expensive process (Lahaye and Ray 1996; Lahaye 1998; Kraan 2012). The methods employed in the conversion of algal biomass to utilisable compounds are usually chemical, thermochemical or enzymatic (Chen et al. 2015). Chemical and thermochemical methods are performed under extreme conditions such as high temperatures, often with pH extremes and may generate inhibitors, affecting fermentation in downstream applications (Ra et al. 2013; Meinita et al. 2015). Typically, with high-energy demands, these methods also make the process expensive. Biochemical conversion using enzymes has lower energy demands and does not generate inhibitor compounds, but the cost and availability of the enzymes can be a deterrent (Lange et al. 2020). The discovery of novel polysaccharide saccharifying enzymes are required to improve the efficiencies of an enzymatic biomass processing system. Given the marine origin of *Ulva* spp*.* halotolerance of isolated enzymes in addition to thermal stability would be key to their use in valorisation of the biomass.

Research on ulvan saccharifying enzymes has been on the rise in the past decade. An ulvan lyase from *Nonlabens ulvanivorans*, isolated from the faeces of the sea slug *Aplysia punctata*, was the first one to be characterised (Nyvall Collén et al. 2011). The enzyme produced by this Gram-negative organism was atypical compared with others in the database and was assigned a new family of its own. Additionally, a novel unsaturated β-glucuronyl hydrolase from the same organism was identified and characterised (Nyvall Collén et al. 2014). Unsaturated β-glucuronyl hydrolase helps remove the unsaturated ends created by the action of lyases and leaves the polysaccharide amenable to the action of other enzymes required for the complete saccharification of ulvan. Next, novel ulvan lyases from three Alteromonadales from the phylum Proteobacteria were discovered (Kopel et al. 2016). These bacteria had no homology to the previously reported ulvan lyases, were aerobic and isolated using conventional media. Several other ulvan lyases from organisms such as *Formosa agariphila, Glaciecola* sp*., Pseudoalteromonas* sp*., Catenovulum maritimum*, and *Thalassomonas* sp*.,* have been identified and characterised since then and classified into five different CAZy families- PL24, PL25, PL28, PL37 and PL40 (Konasani et al. 2018; Qin et al. 2018; Li et al. 2020; Mondal and Ohnishi 2020; Xu et al. 2021; Wang et al. 2022; Tang et al 2023). Ulvan lyases are the first enzymes that act on ulvan thereby breaking down the polysaccharide to smaller oligomers and creating a unsaturated end in the process. The resulting oligosaccharides are further acted upon by unsaturated glucuronyl hydrolases that recognizes the unsaturated end and cleaves it, thus making the oligosaccharide amenable to hydrolysis by other enzymes. Ulvan being a sulfated polysaccharide also requires the action of sulfatase for removal of sulfate groups that enables other enzymes such as rhamnosidases and xylosidases to act on the oligosaccharides (Foran et al. 2017; Reisky et al. 2019).

In this study we explored the genome of *Vibrio* sp*.* FNV38. More than 100 *Vibrio* species have been identified to date and they are ubiquitous gram-negative bacteria, found in a wide range of marine and aquatic temperate habitats (Flütsch et al. 2023). *Vibrio* FNV38 was identified as part of a screening activity undertaken to mine organisms associated with *Ulva* thalli for ulvan and cellulose saccharifying enzymes (Rodrigues et al. 2017) where it was shown to have activity on these substrates. Here, we confirm the lyase activity of a putative ulvan lyase present in the genome and biochemically characterise the recombinant enzyme.

## Materials and methods

### Whole genome sequencing and bioinformatics analysis

The genomic DNA of the isolate was extracted with a Fast Spin kit for soil (MP Biomedical, USA) as per manufacturer's protocol using an overnight culture grown in Difco Marine Broth 2216 (USA). The genomic DNA was quantified using a Qubit version 2.0 fluorometer (Life Technologies, UK). Quality of the genomic DNA was checked on a 0.8% (w/v) agarose gel. The DNA samples were then sent to MicrobesNG in Birmingham, UK, for sequencing. Here, the genomic libraries were prepared using the Nextera XT library prep kit (Illumina, UK) using 2 ng of DNA as a template. PCR was performed as per manufacturer’s protocol with an increase in the elongation step to 1 min. Libraries were sequenced using an Illumina MiSeq platform and 2 × 250 bp paired end reads. The reads were adaptor and quality-trimmed by MicrobesNG using Trimmomatic-0.30 (Bolger et al. 2014) and were de novo assembled into contigs using SPAdes version 3.7 (Bankevich et al. 2012). Annotation of genes was performed with Prokka v1.6 (Rapid Prokaryotic Genome Annotation) (Seemann 2014). CAZY analysis of the genome sequences was carried out in-house using dbCAN HMMdb v11 (Yin et al. 2012). This Whole Genome Shotgun project has been deposited at DDBJ/ENA/GenBank under the accession JAVLUB000000000. The version described in this paper is version JAVLUB010000000.

### Phylogenetic tree analysis and amino acid sequence alignments

The sequences of the Polysaccharide Lyase family 24 were retrieved from the CAZy database (http://www.cazy.db/PL24.html) (Drula et al. 2022). The sequences retrieved from CAZy and PL24 ulvan lyase isolated from *Vibrio* sp*.* FNV38 were aligned using ClustalO with default parameters (Sievers et al. 2011). Finally, the phylogenetic tree was obtained by neighbour joining (Saitou and Nei 1987) using the BLOSUM62 substitution matrix (Henikoff and Henikoff 1992) and 50 bootstrapping rounds.

### Structure modelling of the enzyme

The structural model of the PL24 ulvan lyase isolated from *Vibrio* FNV38 was derived by homology modelling using M4T (Fernandez-Fuentes et al. 2007a, b) as follow. The crystal structures of a lyase from *Alteromonas* sp. (Qin et al. 2020) (PDB id: 6JQ9) and a ulvan lyase from *Catenovulum maritimum* (Xu et al. 2021) PDB id: 7DRQ) were used as templates. The average coverage and sequence identity between PL24 ulvan lyase isolated from *Vibrio* FNV38 and templates was > 90 and 50% respectively, hence well within the confidence boundaries of comparative modelling (Baker and Sali 2001) The quality and stereochemistry of the model was assessed using Prosa-II (Sippl 1993) and PROCHECK (Laskowski et al. 1993) respectively.

### Cloning of gene

The gene containing the PL24 CAZy domain was codon optimised for expression in *Escherichia coli* and synthesised at GenScript USA Inc using a proprietary gene synthesis technology in a pUC57 vector (Protein and codon optimised gene sequence in supplementary data S1 and S2, gene sequence before codon optimisation- GeneBank OQ992772). The sequence coding for the signal peptide was identified using SignalP 5.0 (Almagro Armenteros et al. 2019) and the gene was amplified without the signal peptide using the following primers with Nhe I and Xho I restriction sites respectively: Forward- 5′-AGTAGCTAGCATGAGCGTGACCCTGGAGAGC-3′ Reverse- 5′-AGCACTCGAGAATATCCAGGTCGATAACTTGCAGGTGC-3′. The PCR products and pET 28a + vector were digested using Nhe I and Xho I (NEB, UK), followed by ligation using T4 DNA Ligase (NEB) to link the vector to the PCR products. The recombinant vector was transformed into competent *E. coli* DH5α (Invitrogen, Fischer Scientific, UK) for maintenance, followed by transformation into *E. coli* BL21 (DE3) (NEB) for the overexpression of the gene.

### Overexpression of recombinant PL24 ulvan lyase

For overexpression, 600 mL Luria Broth (Melford Laboratories, UK) supplemented with 50 μg mL^−1^ kanamycin (Melford Laboratories) was inoculated from an overnight culture. The culture was grown at 37 °C and 180 rpm until the OD_600_ reached 0.6–0.8. Protein expression was then induced with 1 mM IPTG (Merck, Germany) followed by the incubation of the cultures at 16 °C and 180 rpm for 18 h. Cells were harvested by centrifugation (8,500 × *g*, 4 °C, 15 min) and the cell pellets were frozen at − 20 °C until further use. Samples (1 mL) were taken before the addition of IPTG and at the time of harvest and analysed using SDS-PAGE with a SurePAGE Bis–Tris 10% gel (GenScript Biotech, UK) for confirmation of overexpression.

### Purification of recombinant PL24 ulvan lyase

Frozen cells were resuspended in cold 10 mM Tris–HCl buffer (pH 8.5) containing 150 mM NaCl and 5 mM imidazole. Cell disruption was carried out using 5 cycles of sonication (30 s sonication, 10 amplitude and 1 min on ice). The lysate and cell debris were separated by centrifugation (8,500 × *g*, 4 °C, 20 min). Purification of the recombinant enzyme was carried out using affinity chromatography. For this, the supernatant was loaded on Ni–NTA resin (Qiagen, UK) held in a Econo-Pac Chromatography Column (Bio-rad, UK). The column was then washed with a buffer containing 20 mM imidazole, 10 mM Tris–HCl (pH 8.0) and 150 mM NaCl. Elution was carried out using a buffer containing 250 mM imidazole, 10 mM Tris–HCl (pH 8.0) and 150 mM NaCl. Eluted enzymes were diafiltered against 100 mM Tris–HCl buffer (pH 8.5) and concentrated using a 10 kDa macrosep advanced centrifugal device (Pall, UK). Purification fractions were analysed using BCA Protein Assay Kit (Thermo Fischer Scientific, UK).

### Ulvan lyase assay

Ulvan lyase activity was estimated by analysing the formation of unsaturated ends or double bonds at 235 nm, which is characteristic of polysaccharide lyase enzymes. Ulvan was extracted from bladed *Ulva* biomass consisting of a mix of *Ulva rigid*a and *Ulva compressa* collected from Milford Haven estuary, Wales, UK at OS reference SN 01767 07627. Extraction was carried out using an aqueous solution of sodium oxalate as described previously (Robic et al. 2009). The assay mix was prepared as described previously (Nyvall Collén et al. 2011). Briefly, it consisted of a 1 mg mL^−1^ solution of ulvan in assay buffer (100 mM Tris, 200 mM NaCl, pH 8.5). An 18 ng aliquot of enzyme was added to 1.2 mL of assay mix, incubated for 10 min, heat killed (95 °C for 10 min), and the absorbance was read at 235 nm. For the control, heat killed enzyme was added to the reaction mix and treated as above. All assays were carried out in triplicate.

### Influence of temperature

To determine the optimal reaction temperature, the ulvan lyase assay was conducted as above except the enzyme was added to pre-heated assay mix and incubated for 10 min at temperatures between 15 °C-60 °C at 5 °C intervals. The influence of temperature on enzyme stability was assessed by incubating the enzymes at temperatures between 15 °C to 60 °C at 5 °C intervals for 2 h. The enzymes were assessed for residual activity as described above at the optimal temperature of 30 °C.

### Influence of pH and NaCl concentration

To evaluate the influence of pH on enzyme activity, the ulvan lyase assay was conducted as above except 100 mM buffers with 200 mM NaCl were used with citric acid-sodium citrate buffers from pH 5.0 to 6.0, sodium phosphate buffers from pH 6.0 to 7.5, Tris–HCl buffer from pH 7.5 to 9.0 and glycine–NaOH buffers from pH 9.0 to 10.0. To evaluate the influence of NaCl concentration on the activity, 100 mM Tris–HCl at pH 8.5 was used with different NaCl concentrations ranging from 0–3 M. Enzyme activity was determined as described above at the optimal temperature of 30 °C.

### Influence of metal ions and small molecules

To determine the effect of metal ions and small molecules on enzyme activity, the ulvan lyase assay was conducted as above except 0.1 mM final concentrations of ZnCl_2_, NiCl_2_, MnCl_2_, MgCl_2_, CaCl_2_, CoCl_2_, CuCl_2_, FeSO_4_, KCl, EDTA and DTT were added individually to the reaction mix and the activity was assessed as described above.

### Kinetic measurements

Assay mixtures containing a final ulvan concentration between 0.05 and 2 mg mL^−1^ in 100 mM Tris–HCl pH 8.5 and 0.6 M NaCl were used to determine enzyme kinetics. Reactions were carried out at 30 °C in a spectrophotometer with a temperature regulated cuvette holder. A total of 18 ng of purified enzyme was mixed with 1.2 mL of the substrate solution and the increase in absorbance at 235 nm was measured over 3 min at 15 s intervals and used to calculate the formation rate of the unsaturated glucuronyl residue using the extinction coefficient of 4800 M^−1^ cm^−1^ (Nyvall Collén et al. 2014). K_m_ and V_max_ values were calculated using a Michaelis–Menten plot in R Studio.

### Analysis of breakdown product

A 5 mg mL^−1^ solution of ulvan was prepared in 100 mL of 100 mM Tris–HCl pH 8.5 and 0.6 M NaCl. Hydrolysis reactions were carried out overnight at 20 °C and 100 rpm for 24 h with 52 µg of enzyme. The reaction mixture was topped up with the same amount of enzyme after 15 h. Breakdown of the polysaccharide was verified by comparative analysis of unhydrolysed and hydrolysed samples using C-PAGE as described by Zablackis and Perez (1990).

Identification of the major breakdown products was achieved by High Pressure Anion Exchange Chromatography (HPAEC). A small portion (25 µL) of the reaction mixture was injected on a Thermo ICS6000 chromatographic system composed of an AG11-HC guard column (4 × 50 mm) and an AS11-HC anion exchange column (4 × 250 mm) connected in series and equilibrated in 8 mM NaOH. Elution was at a flowrate of 0.5 mL min^−1^ using a multistep NaOH gradient from 8 to 280 mM in 40 min. Charged oligosaccharides were detected using a DC conductivity detector associated to a DRS600 suppressor functioning under a 347 mA current. For comparison, previously purified and characterised oligomers (Δ-Rha3S, Δ-Rha3S-HexA-Rha3S, Δ-Rha3S-Xyl-Rha3S) from ulvan (Reisky et al. 2019) were also injected.

## Results

### Whole genome sequencing and bioinformatics analysis

The genome of *Vibrio* sp*.* FNV38 (Accession number JAVLUB010000000) consists of 6,617,998 base pairs and has a GC percentage of 44.58%. The N50 of the assembly is 16,458 and the mean coverage is 21.94X. Analysis of the whole genome resulted in the identification of several CAZymes that can metabolise different algal polysaccharides such as ulvan (PL24, PL25, GH 43, GH78, GH88, GH3, GH39, GH2), cel lulose (GH1, GH3, GH9), agar (GH50, GH117), and alginate (PL6, PL7, PL15, PL17). Supplementary Table 1 gives a detailed split of the individual CAZy families and the number of genes corresponding to each individual family. Additionally, several sulfatases are also present in the genome. Notably, the genome of *Vibrio* FNV38 has several enzymes involved in the breakdown of ulvan including two ulvan lyases (PL24 and PL25), an unsaturated glucuronyl hydrolase (GH 88), a rhamnosidase (GH78), four xylosidases (All GH43), and two S1_7 sulfatases that are known to act on sulphated xylose in ulvan.

### Phylogenetic tree analysis and amino acid sequence alignments

**Fig. 2:**
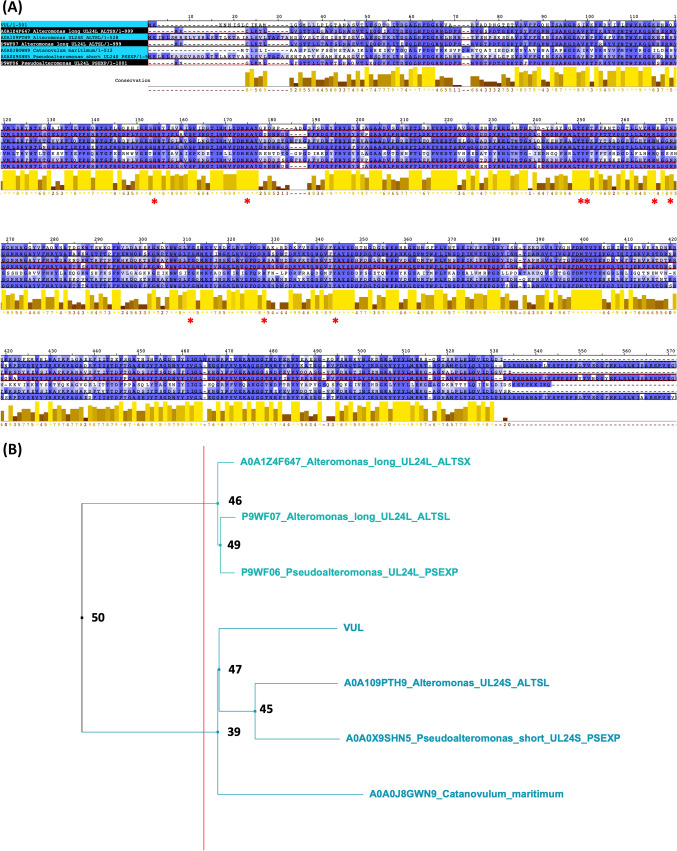
Alignment and phylogenetic tree of the PL24 ulvan lyase isolated from *Vibrio* sp*.* FNV38. PL24 ulvan lyase isolated from *Vibrio* sp*.* FNV38 is represented as ‘VUL’ in the tree; the rest of lyase are labelled following CAZy DB annotation (http://www.cazy.org/PL24_characterized.html). **A**. Alignment of ulvan lyase sequences. Each position of the alignment is coloured according to the conservation from dark blue (conserved) to white (non-conserved). The conservation is also shown as a bar plot in the bottom of the alignment. Red “*” marks indicate catalytic residues as described in (Xu et al. 2021). **B**. Phylogenetic tree; the vertical red line define the distance (as computed using BLOSUM62) separating the two major groups on the PL24 family. Values at the branching points indicate the bootstrap values (out of 50 rounds)

The sequence of PL24 ulvan lyase identified in *Vibrio* FNV38 was aligned to the validated sequences of the ulvan lyases from PL24 family extracted from the CAZy database and a phylogenetic tree was derived. As shown in Fig. 2, the PL24 ulvan lyase from *Vibrio* FNV38 clusters together with the shorter variants of PL24 ulvan lyase family. In particular, it clusters with both *Alteromonas* sp*.* (Uniprot id. A0A109PTH9) and *Pseudoalteromonas* sp*.* (Uniprot id: A0A0X9SHN5) ulvan lyases as well as *Catanovulum maritium* (Uniprot id A0A0J8GWN9). In a different group, all the three annotated sequences of the '*long*' version of the ulvan lyase of family PL24 conformed to the second group. Thus, it can be said the PL24 ulvan lyase isolated from *Vibrio* FNV38 belongs to the PL24 ulvan lyase short variants.

### Structure modelling of the enzyme

**Fig. 3:**
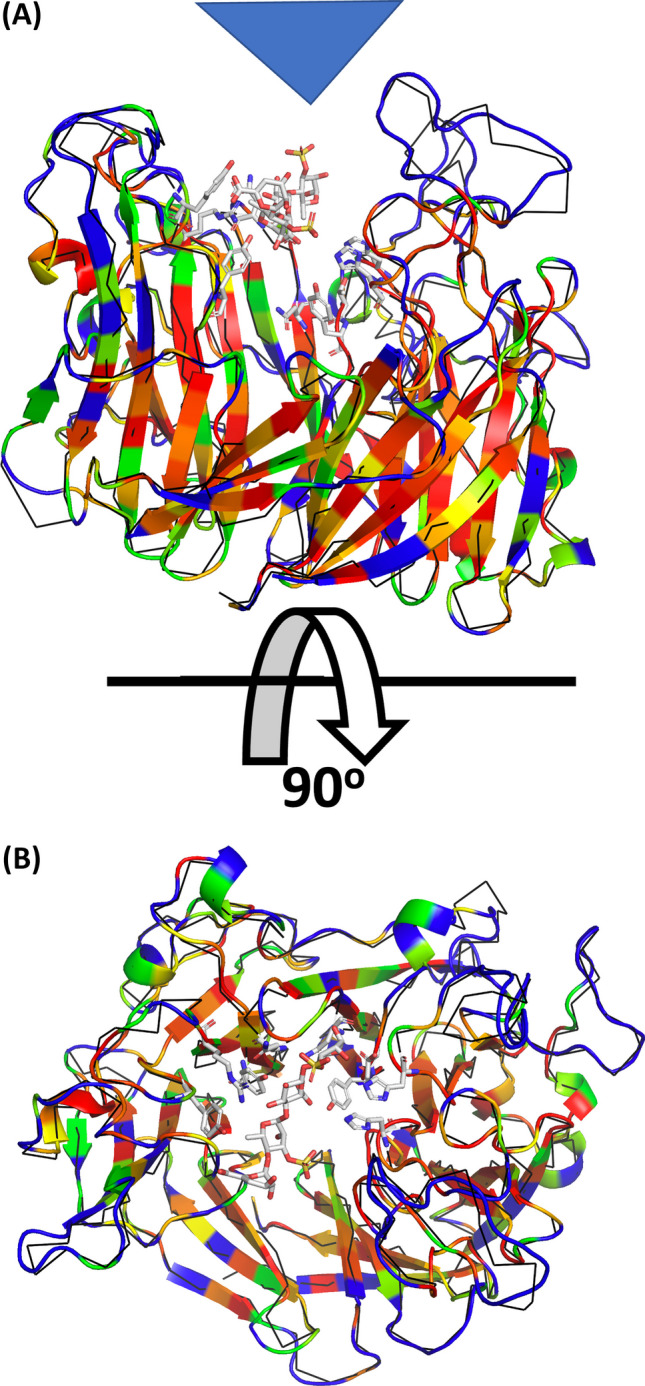
Structural model of the PL24 ulvan lyase isolated from *Vibrio* sp*.* FNV38. Structure rendered in cartoon representation and coloured according to the conservation: red (conserved) to blue (non-conserved). **A**: lateral view with active site location shown with a blue triangle; **B**: top view. The structural superposition of the of the ulvan PL24 from bacterial *Alteromonas sp.* (PDB code: 6byx) is also shown in black ribbon representation bound to the tetrasaccharide substrate shown in stick representation. The catalytic residues as described in (Ulaganathan et al. 2018) are also shown in stick representation

The structure of PL24 ulvan lyase isolated from *Vibrio* FNV38 was modelled against structures of characterized ulvan lyases from *Alteromonas* sp*.* (PDB no.6JQ9) and *Catanovulum maritimun* (PDB no.7DRQ). The novel protein exhibits a fold of a β-propeller with seven blades. Each blade consists of four anti-parallel β-strands with some of these connected by short 3^10^ helices (Fig. 3). As described previously (Xu et al. 2021), the catalytic site of PL24 lyases lies on the top cavity of the propeller flanked by long loops (Fig. 3A). The mapping of the conservation reveals an important conservation mainly on the β-strands conforming the inner regions of the β-propeller as well as the region conforming the active site (Fig. 3B).

### Cloning, overexpression and purification of recombinant ulvan lyase

Analysis of the gene sequence revealed that the first 27 amino acids at the N-terminal of the protein coded for a signal peptide, therefore cloning was carried out by excluding the first 81 base pairs of the gene sequence. *E. coli* BL21(DE3) was used for the expression of the ulvan lyase and SDS-PAGE was used to verify expression of the gene. A poor expression of the enzyme was observed with no noticeable band for the induced enzymes seen on the gel; however, the cell lysate was analysed as during the assay to monitor double bond formation at 235 nm, and enzyme activity was detected in the lysate. Therefore, the purification and characterisation of the enzyme was conducted. Purification of the recombinant enzyme was carried out using Ni–NTA resin and a single band was observed for the purified fraction (Supplementary Fig. 1). The activity of the purified enzyme was confirmed as described above.

### Influence of temperature

**Fig. 4:**
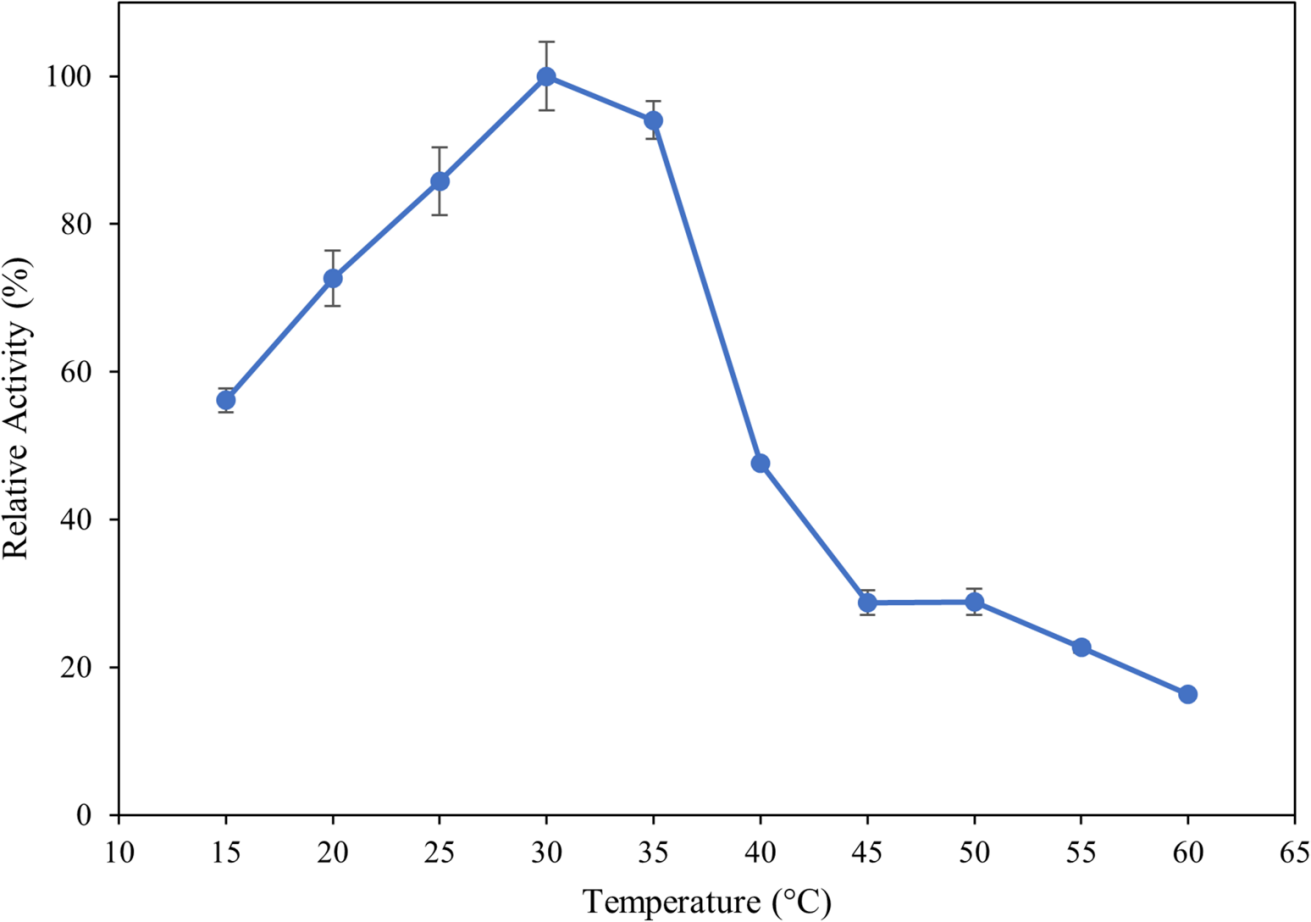
Influence of reaction temperature on enzyme activity. The reaction was performed at temperatures from 15 °C to 60 °C for 10 min, stopped by heat-killing the enzyme and measuring the absorbance at 235 nm. Error bars represent standard deviation of triplicates

**Fig. 5:**
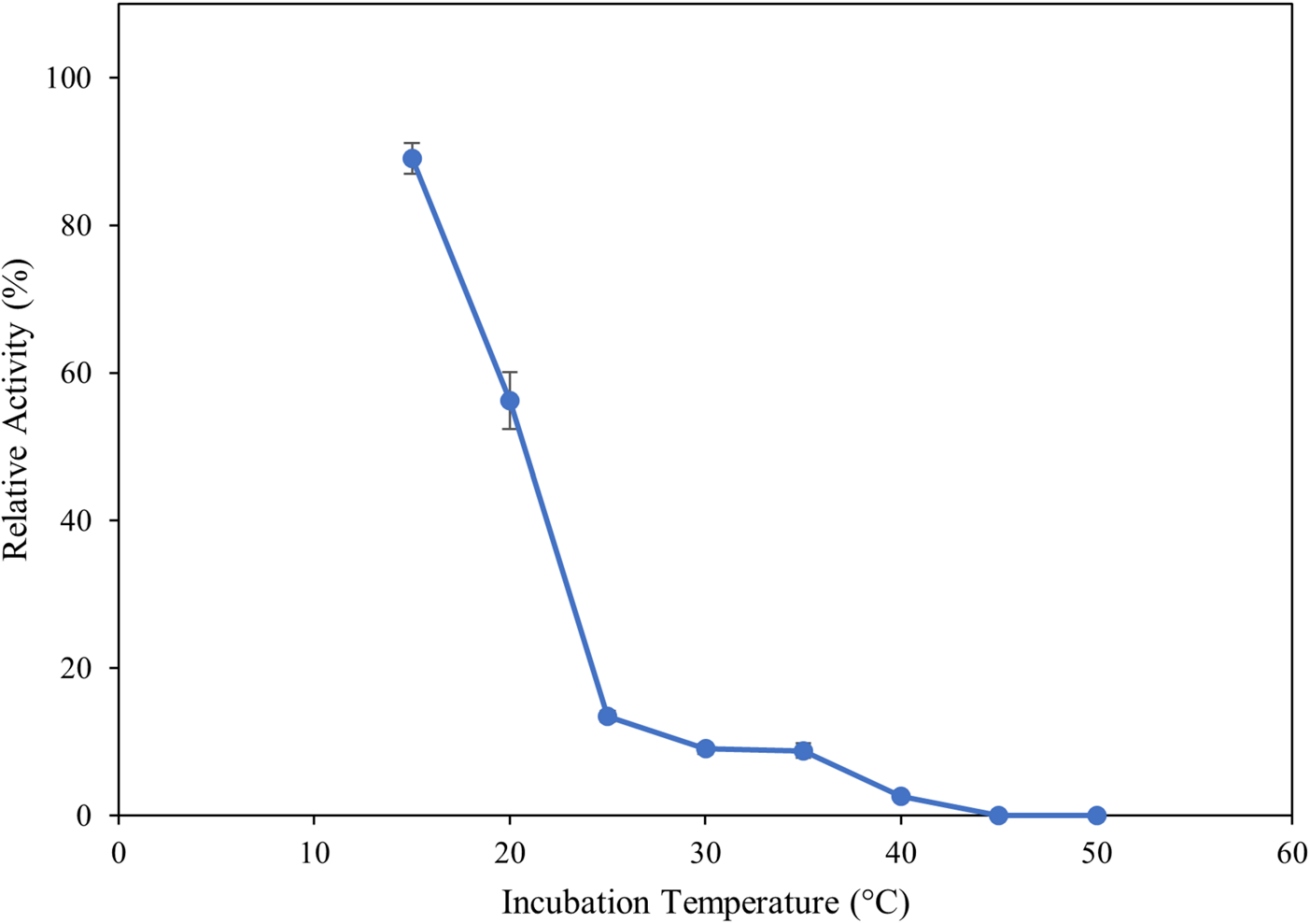
Influence of temperature on enzyme stability. The enzyme was incubated at temperatures ranging from 15 °C to 50 °C for two hours. Enzyme activity was then assessed and compared to initial enzyme activity before stability incubation to determine residual enzyme activity. Error bars represent standard deviation of triplicates

The influence of temperature on the activity and stability of the recombinant ulvan lyase was investigated in the range of 15 °C to 60 °C. Enzyme activity steadily increased until 30 °C, decreased slightly at 35 °C, and then significantly decreased thereafter (Fig. 4). Further, the stability of the enzyme was tested between the same temperature range. The enzyme is highly unstable with up to 45% loss of activity after two hours of incubation at 20 °C and 90% loss of activity after two hours of incubation above 25 °C (Fig. 5).

### Influence of pH and NaCl concentration

**Fig. 6:**
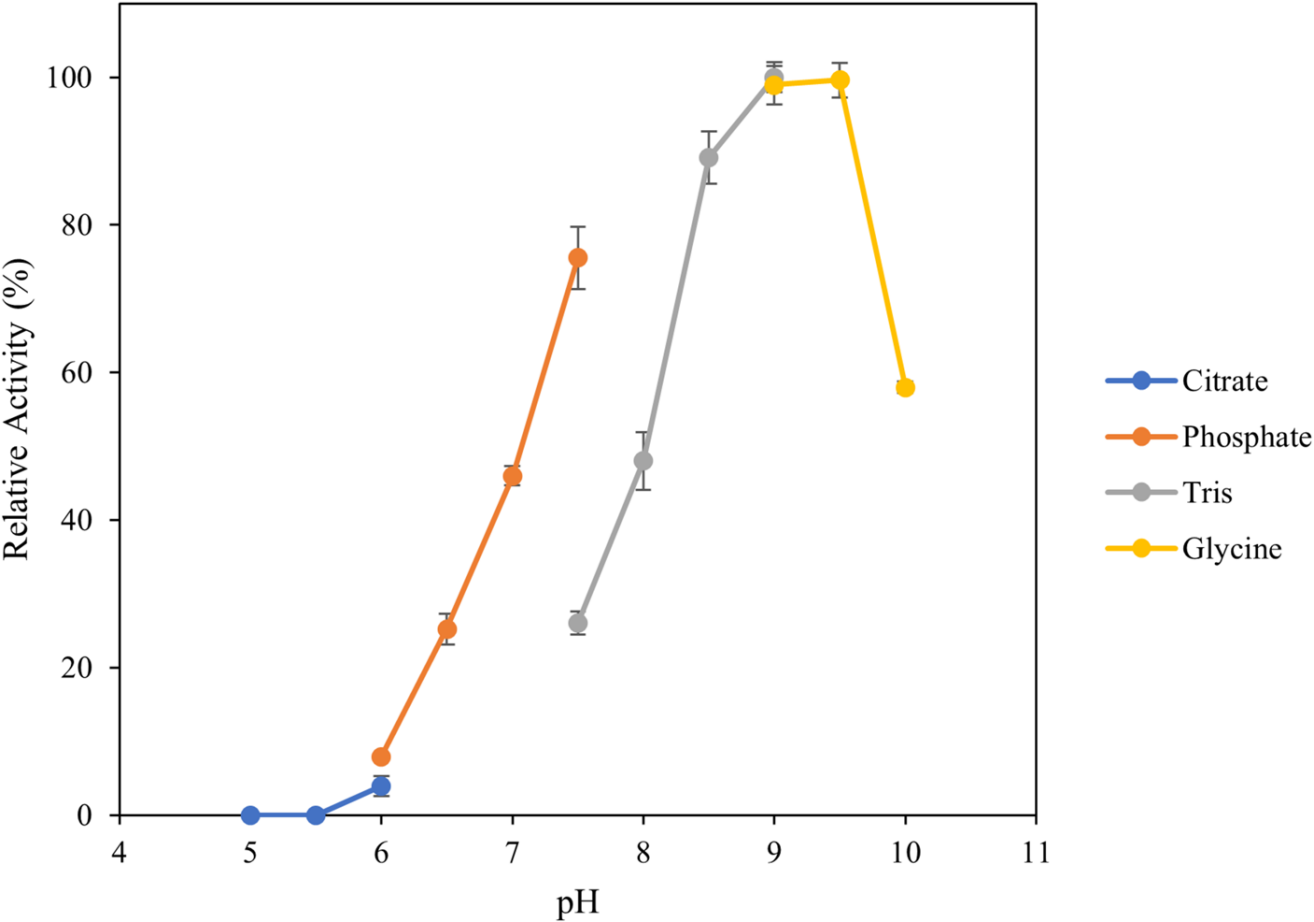
Determination of pH optima. The reaction was performed using different pH and buffer types with some overlaps at 30 °C for 10 min, stopped by heat killing the enzyme and measuring the absorbance at 235 nm. Error bars represent standard deviation of triplicates

In order to determine the optimum pH for enzyme activity, activity was assessed in a range from pH 5.0 to pH 10.0 (Fig. 6). Additionally, similar overlapping pH with different buffer types were tested to determine the influence of buffer species on enzyme activity. Optimal activity was observed between pH 9.0—9.5. No difference in Tris–HCL and glycine–NaOH at pH 9.0 was observed. However, a significant difference in activity between sodium phosphate and Tris–HCl buffers at pH 7.0 was observed. This shows that not only the pH, but also the buffer type can influence enzyme activity.

**Fig. 7:**
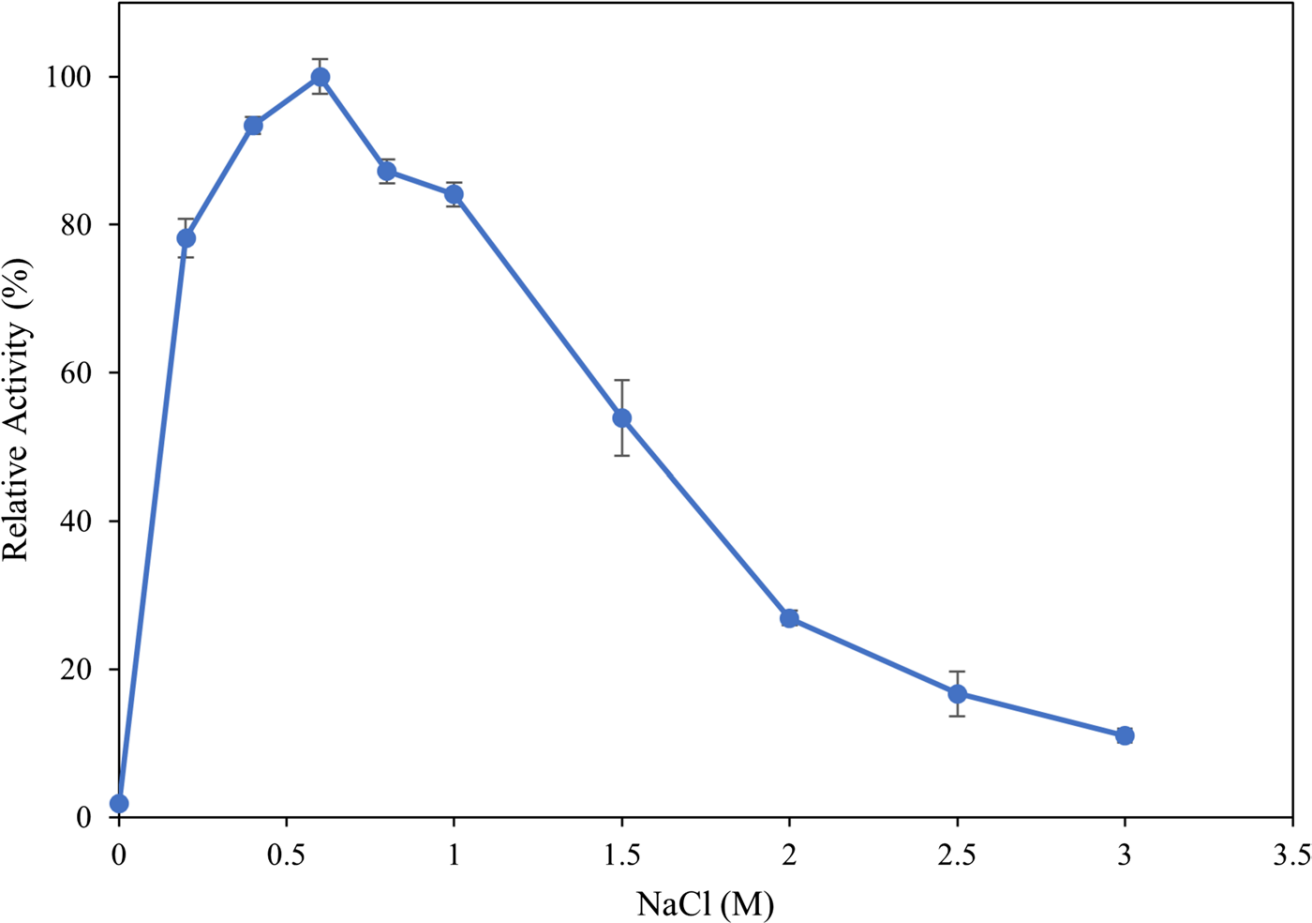
Determination of NaCl optima. The reaction was performed using different NaCl concentration between 0 and 3 M NaCl for 10 min, stopped by heat killing the enzyme and measuring the absorbance at 235 nm. Error bars represent standard deviation of triplicates

Since *Vibrio* FNV 38 was isolated from a marine environment, the influence of NaCl concentration on the enzyme was also evaluated (Fig. 7). Presence of NaCl in the reaction significantly increased enzyme activity with up to 80% increase in the presence of 0.2 M NaCl compared to the absence of NaCl. Enzyme activity increased up to 0.6 M NaCl after which it gradually decreased with significant loss of activity above 1.5 M NaCl. Therefore, 0.6 M is the optimum NaCl concentration for the activity of this enzyme.

### Influence of metal ions and small molecules

**Fig. 8:**
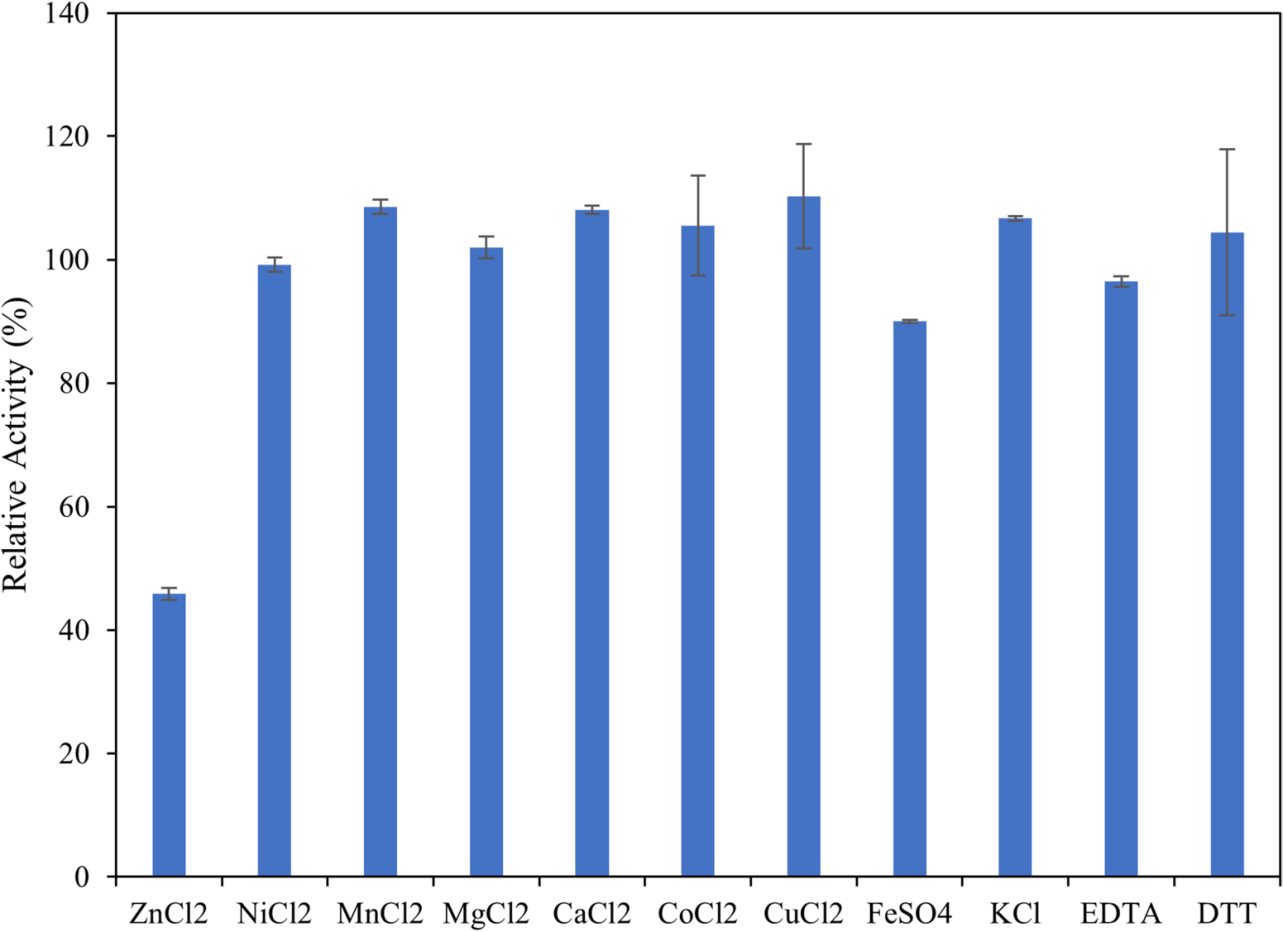
Influence of metal ions and small molecules on enzyme activity. Reactions were carried out by adding 0.1 mM final concentration of different metal ions and small molecules in the reaction mix. Error bars represent standard deviation of triplicates

Given that sea water is a complex mix of different metal ions, the influence of metal ions on enzyme activity was investigated. Additionally, EDTA was tested to determine the dependency of the enzyme on metal ions (Fig. 8). The addition of metal ions in the reaction mix did not significantly improve enzyme activity with a maximum increase of 10% observed in the presence of CuCl_2_. Similarly, the presence of EDTA did not result in any significant loss of activity indicating that the enzyme is unaffected by the presence of metal ions.

### Enzyme kinetics

**Fig. 9:**
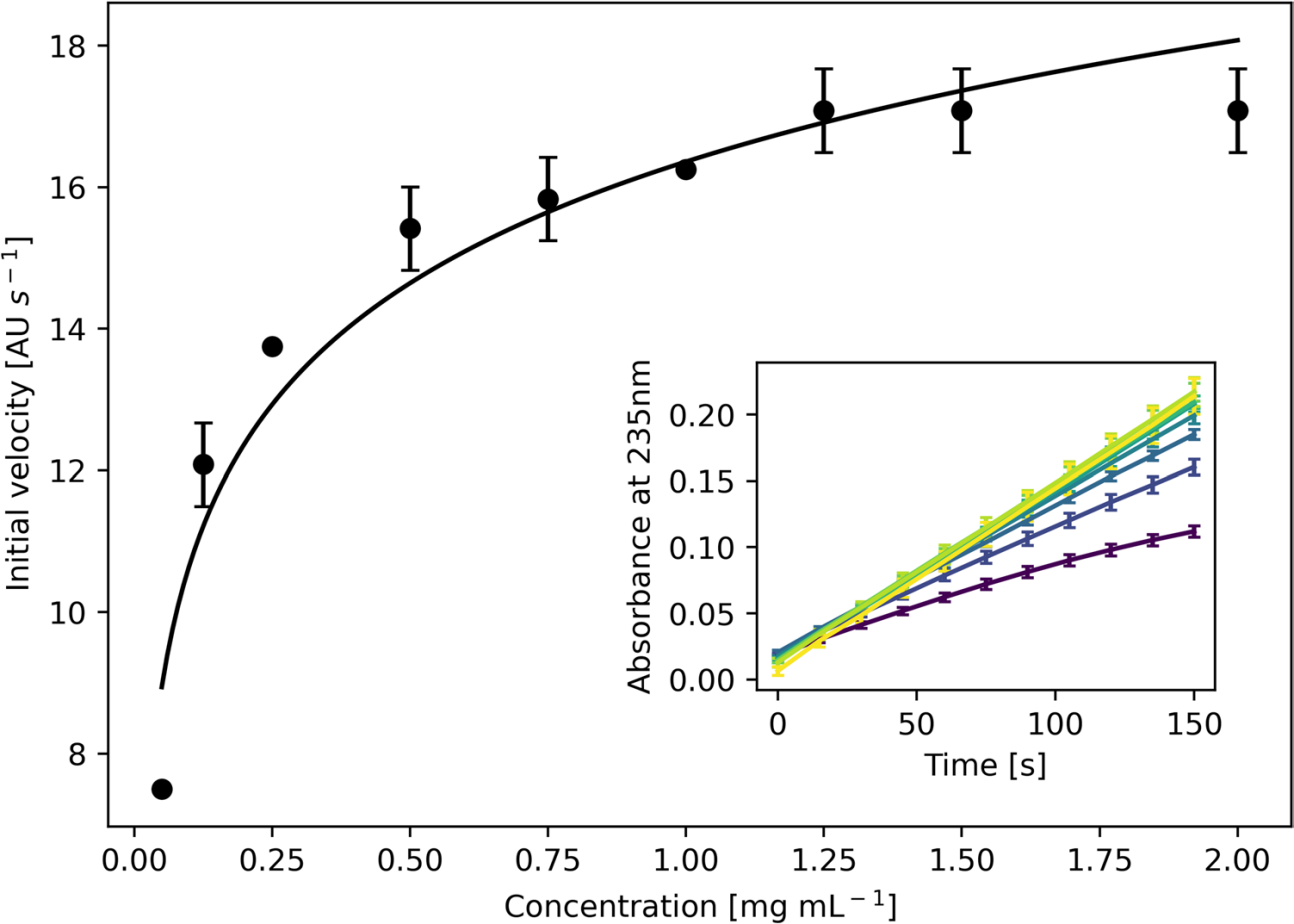
Kinetics of PL24 ulvan lyase from *Vibrio* sp. FNV 38. Michaelis–Menten plot of ulvan degraded by the enzyme. Inset shows linear regression of the different ulvan concentrations from 0.05 mg mL^−1^ (*purple*) to 2 mg mL^−1^ (*yellow*)

The kinetic parameters for the enzyme were determined using the Michaelis–Menten plot (Fig. 9). The K_m_ of the enzyme was 0.064 ± 0.002 g L^−1^ and the V_max_ is 17.57 ± 0.61 μM min^−1^. The K_cat_ is 22.33 s^−1^ and K_cat_/K_m_ is 348.90 mL mg^−1^ s^−1^. The low K_m_ value indicates a high affinity of the enzyme for ulvan.

### Analysis of breakdown products

**Fig. 10:**
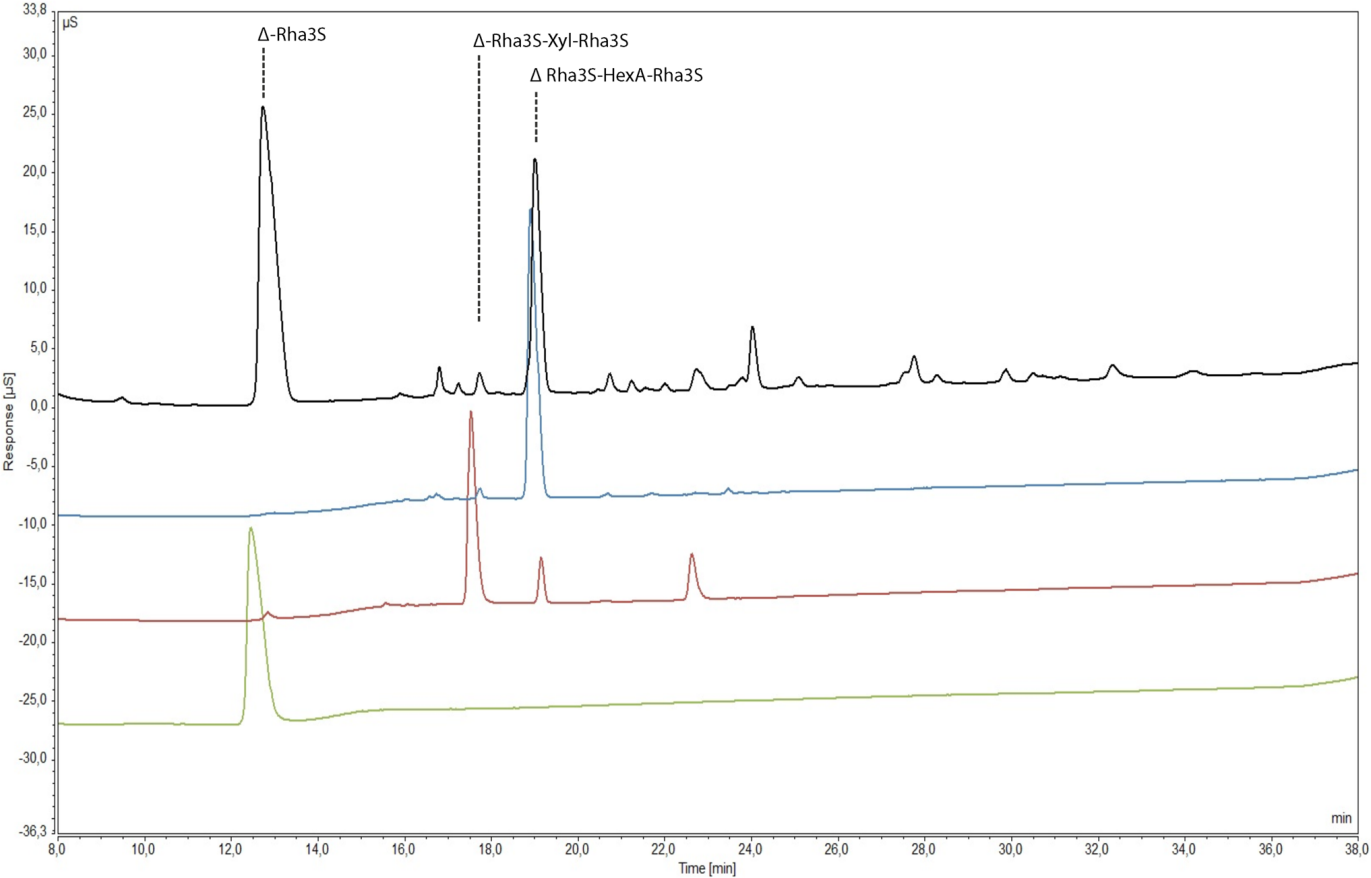
Anion-exchange chromatograms of the oligosaccharides mixture produced by the PL24 ulvan-lyase (black) together with previously purified and characterized standards (Reisky et al. 2019, Δ-Rha3S in green, Δ‑Rha3S‑HexA-Rha3S in red, Δ-Rha3S-Xyl-Rha3S in blue)

The breakdown products resulting from enzymes activity were analysed using C-PAGE and HPAEC. C-PAGE confirmed the breakdown of the polysaccharide into small molecular weight products (Supplementary Fig. 2). HPAEC analysis revealed that these products were predominantly disaccharides and tetrasaccharides. In comparison to standards obtained from previously characterised breakdown products (Reisky et al. 2019), it was determined that these were made up of Δ-Rha3S and Δ-Rha3S-HexA-Rha3S as major products and Δ-Rha3S-Xyl-Rha3S as a minor product (Fig. 10, where Δ represent 4-deoxy-L-threo-hex-4-enopyranosiduronic acid, an unsaturated uronyl residue formed by lyases at the non-reducing end of the products and HexA represents either glucuronic acid or iduronic acid (Hexuronic acids).

## Discussion

*Vibrio* sp*.* FNV38 has a diverse CAZy profile. CAZy or carbohydrate active enzymes, as the name suggests, are enzymes that are involved in metabolising carbohydrates. Glycoside hydrolase, polysaccharide lyases, and carbohydrate esterases are involved in the breakdown of polysaccharides, whereas glycosyl transferases are involved in the synthesis of polysaccharides. Carbohydrate binding modules are a class of CAZymes that are not catalytic in function, but help associated catalytic modules bind to the substrate and those classified as auxiliary activity are redox enzymes that act in conjunction with the catalytic domains (Lombard et al. 2014). Microbes that possess these enzymes in the marine environment play a crucial role in providing carbon sources to other organisms by breaking down the polysaccharides that are available. Although *Vibrio* FNV 38 was isolated from the thallus of *Ulva,* CAZymes in its genome demonstrate its ability to metabolise polysaccharides found in other seaweed in addition to ulvan. Presence of several genes involved in sulphate metabolism in the genome can be attributed to the abundance of sulphated polysaccharides in the marine niche that this organism was isolated from. Additionally, it possesses 47 glycosyl transferases, which leads us to hypothesize that it could be involved in biofilm formation on the algal thallus.

Ulvan is a complex polysaccharide, and several enzymes are required for its saccharification. *Vibrio* FNV 38 has several genes required for all the enzymes involved in ulvan saccharification including ulvan lyases, unsaturated glucuronyl hydrolase, rhamnosidases, xylosidases and sulfatases. Ulvan saccharification has been elucidated in some bacteria including *Alteromonas* sp*.* LOR, *F. agariphila* KMM 3901, and *Glaciecola* sp*.* (Foran et al. 2017; Salinas and French 2017; Reisky et al. 2019; Mondal and Ohnishi 2020). Comparison of the CAZymes and sulfatases in *Vibrio* FNV 38 to these organisms reveals that the overall ulvan saccharifying capability of this organism is comparatively limited. While the three other organisms have both GH88 and GH105 that are involved in the removal of the unsaturated residue after the action of the lyase, *Vibrio* FNV38 only has a GH88. Additionally, although the genome of *Vibrio* has 12 sulfatases, only two belonging to family S1_7 (endo-xylose-2-sulfate-2-O-sulfohydrolase) are capable of acting on ulvan. In comparison, *Glaciecola* sp*.* has a S1_8 (exo-xylose-2-sulfate-2-O-sulfohydrolase) and S1_25 e-rhamnose-3-sulfate-3-O-sulfohydrolase) in addition to S1_7; and *F. agariphila* has four S1_3, two S1_8, S1_25, and S1_27 (ulvan sulfohydrolase). Desulfation of the polymer/oligomer is essential to the action of other enzymes in the cascade and therefore incomplete desulfation of the polymer can limit the organism’s capability to utilise it. However, given that *Vibrio sp.* FNV 38 has CAZymes belonging to PL24, PL25, GH 43, GH78, GH88, GH3, GH39, GH2, it can potentially contribute to the initial steps in the saccharification of ulvan, following which other organisms present in the environment that have the capability to utilise these smaller products could further metabolise the substrate.

A gene coding for a putative PL24 ulvan lyase was cloned in *E.coli* for the production of a recombinant enzyme. The optimum working conditions for the enzyme were determined with the enzyme working optimally at 30 °C. Temperature can often affect the stability of enzymes and therefore, the influence of temperature on this enzyme was assessed by incubating it over a wide range of temperatures over two hours, then assessing its residual activity. The enzyme is highly unstable even at temperatures of 25 °C and strategies to stabilize the enzyme such as mutagenesis need to be undertaken to enable the use of the enzyme in hydrolysis reactions which could take several hours to complete. The enzyme shows appreciable stability at 15 °C and this could be attributed to the psychrophilic nature of the organism it was cloned from. Enzymes that are stable and active at lower temperatures could be beneficial for use in industrial processes in countries with a cooler climate as there would be no heating costs involved in the hydrolysis process.

With regards to the pH, unlike other previously characterised ulvan lyases a typical bell-shaped curve is not observed with almost equal optimal activity being recorded at pH 9.0 and 9.5 in Tris–HCl and glycine–NaOH buffers. However, at lower pH of 7.0 and 7.5 Tris–HCl shows significantly lower activity in comparison to sodium phosphate buffers, highlighting the influence of buffer salts on the activity of the enzyme. This is contradictory to what has been observed for other characterised ulvan lyases, where higher activity has been observed in Tris–HCl compared to sodium phosphate buffers (Reisky et al. 2018; Gao et al. 2019).

Being a marine enzyme, NaCl has a significant influence on the activity of this ulvan lyase, with around 80% increase in activity observed in 0.2 M NaCl compared to the absence of NaCl. The enzyme exhibited around 80% or higher relative activity at concentrations between 0.2 M and 1 M NaCl, with 0.6 M NaCl being optimal. Enzyme activity was inhibited at NaCl concentrations above 1 M. Additionally, the influence of various metal ions that are present in seawater were evaluated on the enzyme. Presence of zinc significantly inhibited the activity of the enzyme, and no metal was found to significantly enhance enzyme activity. Additionally, presence of EDTA had no effect on enzyme further confirming that the enzymes does not depend on metals as a co-factor to function, unlike other ulvan lyases which are Ca^2+^ dependant.

The K_m_ of the enzyme is 0.06 g L^−1^ and the V_max_ is 17.37 ± 0.45 μm min^−1^. A low K_m_ value indicates a high affinity of the enzyme for ulvan. The K_m_ in this case is significantly lower in comparison to previously characterised enzymes such as ulvan lyases isolated from *Thalassomonas* sp*.* LD5 (1.01 mg mL^−1^), *Pseudoalteromonas* (2.1 mg mL^−1^) and *Alteromonas* (7.2 mg mL^−1^) (He et al. 2017; Qin et al. 2018; Wang et al. 2022). These differences in kinetic properties could be due to the differences in the ulvan used, as seen in the case of an ulvan lyase extracted for *F. agariphila*, where the K_m_ of the same enzyme on ulvan extracted from *Ulva armonicana*, *Ulva intestinalis* and *U. lactuca* was 0.7, 3.0 and 1.2 mg mL^−1^ respectively (Konasani et al. 2018). The breakdown products of the enzyme were primarily disaccharides and tetrasaccharides with Δ-Rha3S and Δ-Rha3S-HexA-Rha3S as major products and Δ-Rha3S-Xyl-Rha3S as a minor product. These are similar to the breakdown products obtained by the action of the short ulvan lyase from *Alteromonas*, indicating a similar mechanism of action. Additionally, phylogenetic analysis of the gene sequence, places this lyase with “short type ulvan lyases”, that are characterised by the absence of a conserved type II dockerin repeat domain normally associated with long type ulvan lyases (Kopel et al. 2016). The structure of the enzyme based on the predicted model further helps confirm this as it is like the seven bladed β-propeller of LOR_107 which is short type. Although the optimum working condition of the enzyme in the current study is similar to other short type ulvan lyases in term of the pH and NaCl optima, a significant difference in the temperature optima and stability exists. This could potentially be attributed to temperature adaptations to the different environments that these enzyme containing organisms were isolated from.

In conclusion, this ulvan lyase characterised from *Vibrio* sp*.* further adds to our knowledge and understanding of PL24 ulvan lyases that are present in the marine environment. Its stability and activity at lower temperatures make it a valuable resource which could be further evaluated for use in energy-efficient biorefinery processes for *Ulva* spp*.* in countries with a cooler climate*.*

### Supplementary Information

Below is the link to the electronic supplementary material.

Supplementary file1 (DOCX 11066 KB)

## Data Availability

The datasets generated during and/or analysed during the study are available from the corresponding author on reasonable request.
