# Supplementary material for: Biochemical characterisation of a PL24 ulvan lyase from seaweed-associated Vibrio sp. FNV38
Source: J Appl Phycol. 2023 Dec 7;36(2):697–711. doi: 10.1007/s10811-023-03136-3 (PMC11101340; doi:10.1007/s10811-023-03136-3)
Supplement: Supplementary file 1 — Supplementary file1 (DOCX 11066 KB) [file 10811_2023_3136_MOESM1_ESM.docx]

**Supplementary Data**

**S1. Protein Sequence**

>VUL

MKKNNISLCIKAMLGSMLLLPLGTANASVTLESDTQITDGALHFDGKKVARFADDNGTET

YDYFFGQHISAHGDSIKKYKDYIFLTWYKGGKENRQVMLSRYNTKSGAIKTIEFPHQHTG

FLGQKHIGESHNYISVAISPIDGTIHMLYDMHAYGKDRPADGSFSDDYFRYSYSIAGAAD

VSDNEFTLDKFVKDTSAVSQGENDFKHLSLTGEIDYDSYSGLTYPTFFRNTDGTLLVYMR

KGGNNNGGYVFAQYNATDGKWTEWKQFNVLDAKSHGNDYNWGLYGSMKYVNDKLRVGFQQRAKRDDKFVYQNGVFYAYSDNTNGDGDWKNHRGENMSFPLVNTDEIKVFEPGDYISHTEKDSVHIVQNFDWTVTEKGDIHTISRVKSRDNKRSDFEMVHLHAYKPLGAEEFIITDEFSGATNIHTAGDDIYIIGLNKNGRPYVEKAKGGTNDFERVYEAESGKQFSHGKVHIKDGKLYYYLMERSQGTSRPLHLQVIDLDI

*Residues highlighted in red code for the signal peptide

**S2. Codon Optimized Gene Sequence**

ATGAAGAAAAACAACATCAGCCTGTGCATTAAGGCGATGCTGGGTAGCATGCTGCTGCTGCCGCTGGGTACCGCGAACGCGAGCGTGACCCTGGAGAGCGACACCCAGATCACCGATGGTGCGCTGCACTTCGACGGCAAGAAAGTTGCGCGTTTTGCGGACGATAACGGTACCGAAACCTATGATTACTTCTTTGGTCAGCACATCAGCGCGCACGGCGACAGCATTAAGAAATACAAGGATTACATCTTCCTGACCTGGTACAAGGGTGGCAAAGAGAACCGTCAAGTGATGCTGAGCCGTTATAACACCAAGAGCGGTGCGATCAAAACCATTGAGTTCCCGCACCAGCACACCGGTTTTCTGGGCCAAAAGCACATTGGTGAAAGCCACAACTACATCAGCGTTGCGATCAGCCCGATTGACGGCACCATTCACATGCTGTATGATATGCACGCGTACGGTAAAGACCGTCCGGCGGATGGCAGCTTCAGCGACGATTATTTTCGTTATAGCTATAGCATTGCGGGTGCGGCGGATGTGAGCGATAACGAATTCACCCTGGACAAGTTTGTGAAAGATACCAGCGCGGTTAGCCAAGGCGAGAACGACTTCAAGCACCTGAGCCTGACCGGTGAAATCGACTATGATAGCTACAGCGGCCTGACCTACCCGACCTTCTTTCGTAACACCGACGGTACCCTGCTGGTGTATATGCGTAAGGGTGGCAATAACAACGGTGGCTATGTTTTCGCGCAGTACAACGCGACCGATGGCAAGTGGACCGAGTGGAAACAATTTAACGTTCTGGACGCGAAAAGCCACGGCAACGATTATAACTGGGGTCTGTACGGCAGCATGAAGTATGTGAACGACAAACTGCGTGTTGGTTTTCAGCAACGTGCGAAGCGTGACGATAAATTCGTGTACCAGAACGGCGTGTTCTACGCGTACAGCGACAACACCAACGGTGACGGCGATTGGAAGAACCACCGTGGTGAAAACATGAGCTTCCCGCTGGTGAACACCGATGAGATTAAGGTTTTTGAACCGGGTGACTACATCAGCCACACCGAGAAAGATAGCGTGCACATTGTTCAAAACTTCGACTGGACCGTGACCGAAAAGGGTGATATCCACACCATTAGCCGTGTTAAGAGCCGTGACAACAAACGTAGCGATTTTGAGATGGTGCACCTGCACGCGTACAAACCGCTGGGCGCGGAGGAATTCATCATTACCGACGAATTTAGCGGTGCGACCAACATTCACACCGCGGGCGACGATATCTACATCATTGGTCTGAACAAAAACGGCCGTCCGTATGTGGAGAAGGCGAAAGGTGGCACCAACGACTTCGAACGTGTTTACGAGGCGGAAAGCGGCAAGCAGTTTAGCCACGGCAAAGTGCACATCAAGGATGGTAAACTGTACTATTACCTGATGGAGCGTAGCCAAGGTACCAGCCGTCCGCTGCACCTGCAAGTTATCGACCTGGATATT

M UI I L FT W E

**
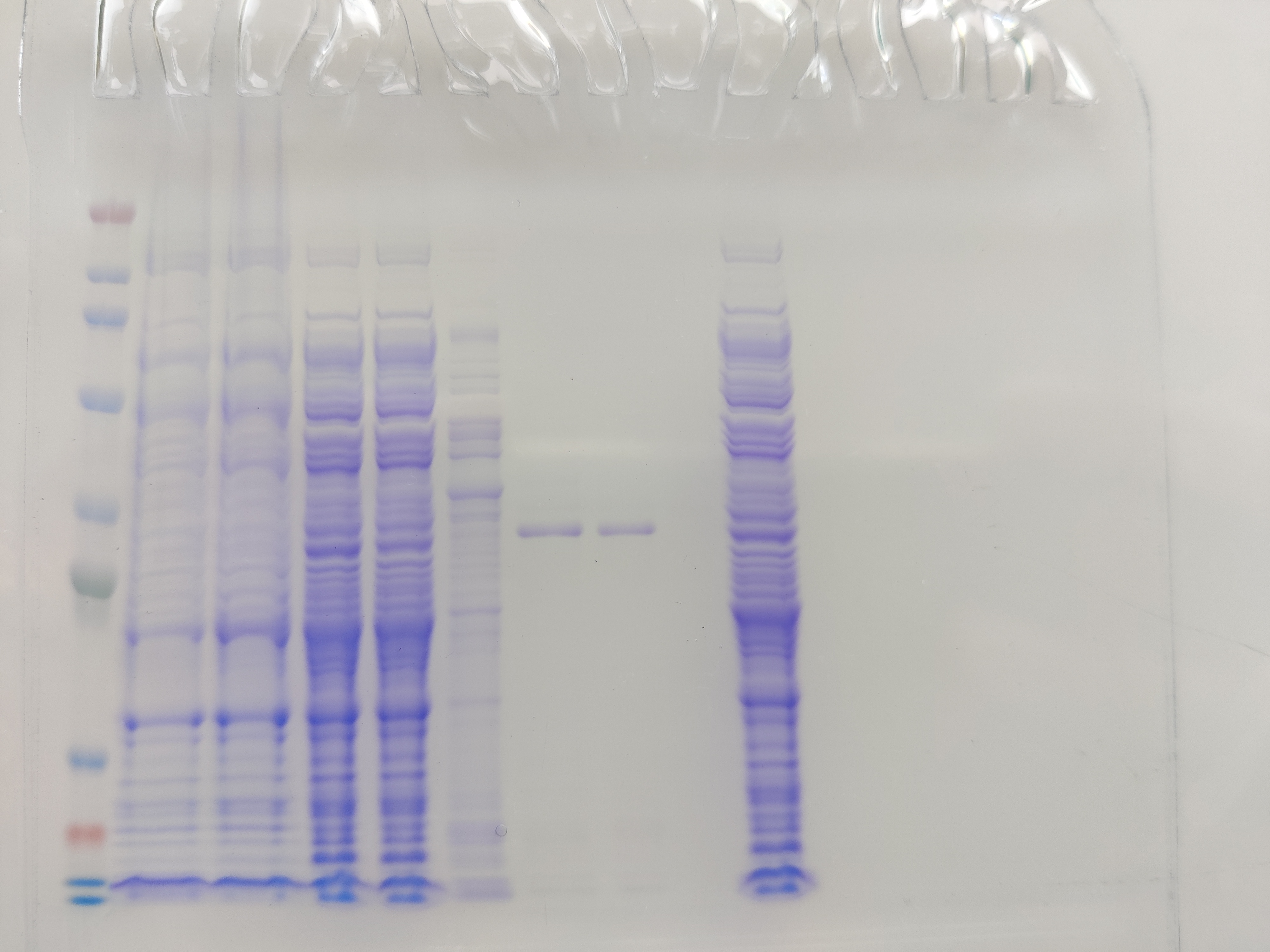
**

270 kDa

175 kDa

130 kDa

95 kDa

60 kDa

50 kDa

35 kDa

30 kDa

**Supplementary Figure 1.** SDS-PAGE of fractions from the purification of recombinant ulvan lyase. Proteins were visualized with Coomassie Brilliant Blue R-250. The theoretical molecular weight of the protein without the signal peptide is 54 kDa Key-M: marker, UI: uninduced, I: induced, L: cell lysate, FT: flow-through, W: wash fractions, E: eluted fraction

A B

**
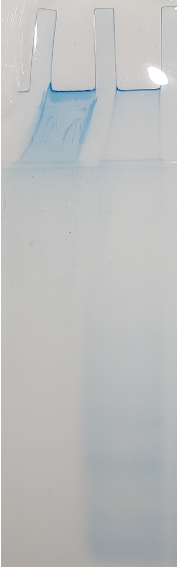
Supplementary Figure 2.** C-PAGE of ulvan before and after saccharification using PL24 ulvan lyase from Vibrio sp. FNV38. Key-A: before saccharification, B: after saccharification.


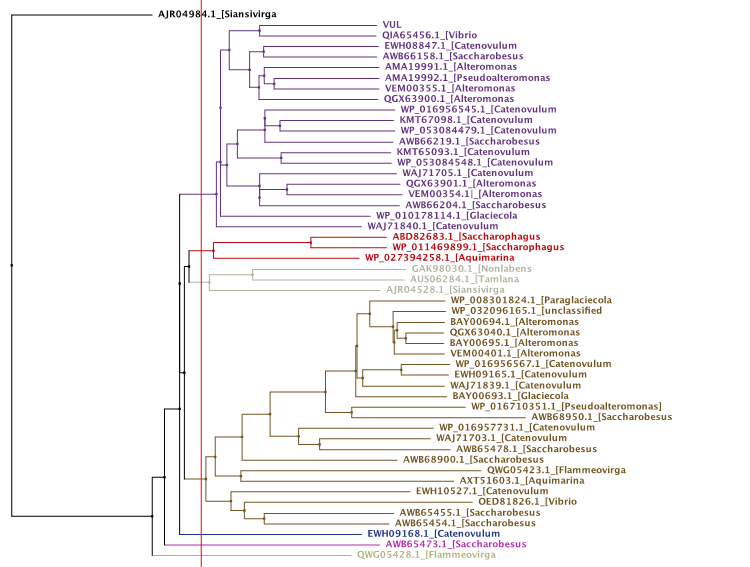


**Supplememtary figure 3.** Phylogenetic tree derived from the multiple sequence alignment of the Polysaccharide Lyase family 24 were retrieved from the CAZy database (http://www.cazy.org/PL24_all.html) composed of 57 members. The sequences are labelled by the Genebank ID followed by organism.

**Supplementary Table 1.** Detailed split of CAZy genes in *Vibrio sp.* FNV 38

| Family | Number of Genes | Family | Number of Genes |
| --- | --- | --- | --- |
| Polysaccharide Lyases | | Carbohydrate Binding Modules | |
| PL 0 | 1 | CBM4 | 1 |
| PL 6 | 2 | CBM5 | 2 |
| PL 7 | 8 | CBM9 | 1 |
| PL15 | 2 | CBM13 | 1 |
| PL17 | 4 | CBM32 | 2 |
| PL 24 | 1 | CBM34 | 1 |
| PL25 | 1 | CBM41 | 1 |
| Glycosyl Hydrolases | | CBM47 | 1 |
| GH1 | 6 | CBM48 | 4 |
| GH2 | 4 | CBM50 | 10 |
| GH3 | 7 | CBM69 | 1 |
| GH4 | 1 | CBM73 | 2 |
| GH5 | 2 | Carbohydrate Esterases | |
| GH9 | 2 | CE0 | 1 |
| GH10 | 2 | CE4 | 1 |
| GH13 | 16 | CE8 | 1 |
| GH16 | 3 | CE9 | 1 |
| GH18 | 3 | CE11 | 1 |
| GH20 | 1 | Glycosyl Transferases | |
| GH23 | 10 | GT1 | 2 |
| GH25 | 1 | GT2 | 11 |
| GH28 | 2 | GT4 | 14 |
| GH36 | 4 | GT5 | 1 |
| GH38 | 1 | GT9 | 3 |
| GH39 | 2 | GT19 | 1 |
| GH43 | 4 | GT25 | 1 |
| GH50 | 4 | GT28 | 2 |
| GH73 | 2 | GT30 | 2 |
| GH77 | 1 | GT35 | 1 |
| GH78 | 1 | GT51 | 5 |
| GH82 | 2 | GT56 | 1 |
| GH88 | 1 | GT58 | 1 |
| GH92 | 1 | GT83 | 1 |
| GH94 | 4 | GT90 | 1 |
| GH102 | 1 | Auxiliary Activities | |
| GH103 | 4 | AA0 | 1 |
| GH116 | 1 | AA10 | 1 |
| GH117 | 2 |  |  |
| GH127 | 1 |  |  |
| GH149 | 1 |  |  |
| GH152 | 1 |  |  |
